# In Vitro and In Vivo Studies of Hydrophilic Electrospun PLA95/β-TCP Membranes for Guided Tissue Regeneration (GTR) Applications

**DOI:** 10.3390/nano9040599

**Published:** 2019-04-11

**Authors:** Chien-Chung Chen, Sheng-Yang Lee, Nai-Chia Teng, Hsin-Tai Hu, Pei-Chi Huang, Jen-Chang Yang

**Affiliations:** 1 Graduate Institute of Biomedical Materials & Engineering, College of Biomedical Engineering, Taipei Medical University, Taipei 110, Taiwan; polyjack@tmu.edu.tw; 2 Ph.D. Program in Biotechnology Research and Development, College of Pharmacy, Taipei Medical University, Taipei 110, Taiwan; 3 School of Dentistry, College of Oral Medicine, Taipei Medical University, Taipei 110, Taiwan; seanlee@tmu.edu.tw (S.-Y.L.); tengnaichia@hotmail.com (N.-C.T.); hsintaihu1011@hotmail.com (H.-T.H.); 4 Department of Dentistry, Wan-Fang Medical Center, Taipei Medical University, Taipei 116, Taiwan; littlepeichi@yahoo.com.tw; 5 Graduate Institute of Nanomedicine and Medical Engineering, College of Biomedical Engineering, Taipei Medical University, Taipei 110, Taiwan; 6 International Ph.D. Program in Biomedical Engineering, College of Biomedical Engineering, Taipei Medical University, Taipei 110, Taiwan

**Keywords:** PLA95, biocompatibility, guided tissue regeneration (GTR), electrospinning

## Abstract

The guided tissue regeneration (GTR) membrane is a barrier intended to maintain a space for alveolar bone and periodontal ligament tissue regeneration but prevent the migration of fast-growing soft tissue into the defect sites. This study evaluated the physical properties, in vivo animal study, and clinical efficacy of hydrophilic PLA95/β-TCP GTR membranes prepared by electrospinning (ES). The morphology and cytotoxicity of ES PLA95/β-TCP membranes were evaluated by SEM and 3-(4,5-cimethylthiazol-2-yl)-2,5-diphenyl tetrazolium bromide (MTT) respectively. The cementum and bone height were measured by an animal study at 8 and 16 weeks after surgery. Fifteen periodontal patients were selected for the clinical trial by using a commercial product and the ES PLA95/β-TCP membrane. Radiographs and various indexes were measured six months before and after surgery. The average fiber diameter for this ES PLA95/β-TCP membrane was 2.37 ± 0.86 µm. The MTT result for the ES PLA95/β-TCP membrane showed negative for cytotoxicity. The significant differences in the cementum and bone height were observed between empty control and the ES PLA95/β-TCP membrane in the animal model (*p* < 0.05). Clinical trial results showed clinical attachment level (CAL) of both control and ES PLA95/β-TCP groups, with a significant difference from the pre-surgery results after six months. This study demonstrated that the ES PLA95/β-TCP membrane can be used as an alternative GTR membrane for clinical applications.

## 1. Introduction

Periodontitis is one of the most destructive diseases that destroys the tooth-supporting tissues, including the alveolar bone, periodontal ligament, and cementum, ultimately leading to tooth loss [1,2,3]. For patients with severe periodontitis, it is critical to remove dental calculus and plaque by scaling and root planning [4]. Guided tissue regeneration (GTR) membranes are typically used to block the migration of fast-growing connective tissue into the bony defect and to create space for the regeneration of slow-growing alveolar bone and periodontal ligament [5]. Over the years, the materials of the GTR barrier matrix from non-resorbable polytetrafluoroethylene-like expanded e-PTFE or dense d-PTFE [6] and titanium mesh [7], evolved to resorbable polymer to dispense with the operation of secondary GTR removal [8,9]. Most commercial resorbable synthetic polymer membranes are based on aliphatic polyesters, such as poly(lactic acid) (PLA), poly(glycolic acid) (PGA), poly(ε-caprolactone) (PCL), or their copolymers, to match the resorption time period for various clinical needs [10]. Among these polymers, a novel copolymer composed of poly-5D/95L-lactide (PLA95) has been successfully used in distal radius fractures [11] and craniomaxillofacial applications like skull flap fixation [12] and facial fracture fixation [13] due to its relatively strong mechanical properties. The feasibility of using PLA95 resorbable GTR membranes is worth exploring.

Due to the potential inflammation risk caused by acid release from the monomer or crystalline debris during degradation which could result in a foreign body immune response [14], bio-ceramics such as β-tricalcium phosphate (β-TCP) and hydroxyapatite (HAp) were used for their pH buffering effects [15] and bone cell response enhancement [16]. In recent years, various osteo-conductive membranes such as polylactic acid (PLA)/HAp [17,18], gelatin/HAp [19], three-layered HAp/collagen/PLGA [20], and nano-apatite/polycaprolactone (PCL) [21] have been fabricated using the electrospinning (ES) technique. These resorbable hybrid membranes help bone reconstruction with calcium ions releasing and good pH buffering properties.

In this study, we prepared the PLA95/β-TCP GTR membranes by ES and dip-coating techniques [22], then the safety and effectiveness of this GTR membrane were assayed by cytotoxicity testing, in vivo, and clinical studies.

## 2. Materials and Methods

### 2.1. ES PLA95/β-TCP Fibrous Membranes

**Figure 1:**
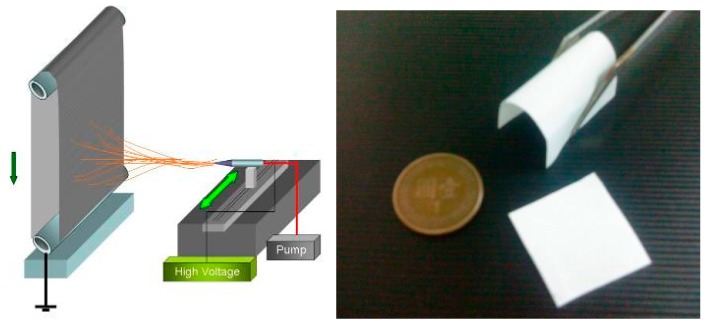
Electrospinning setup and ES PLA95/β-TCP membranes.

Poly-5D/95L-lactide (PLA95) was provided by BioTech One Inc. (Taipei, Taiwan) with inherent viscosity (I.V.) of 0.6 dL/g. The solution dope was prepared by mixing 20 w/v% PLA95 used a mixed dichloromethane/dimethylformamide (DCM/DMF: 7/3 (v/v)) solvent and 3% (w/v) β-TCP powders (<23 μm) under an ultrasonic vibrator to prevent the β-TCP powders from agglomerating, then spun it via electrospinning (ES) technique using the setup shown in Figure 1. 

The ES PLA95/β-TCP membranes were sterilized by gamma-radiation. The membranes were gold-coated, and their morphology was examined by scanning electron microscopy (SEM, S-2400; Hitachi, Tokyo, Japan), followed by characterization with Image J analytical software (NIH, MD, USA)

### 2.2. Cytotoxicity Testing

Cytotoxicity testing was performed using the method described in the ISO-10993-5 guideline. Accordingly, the specimens were divided into the following four groups: test (ES PLA95/β-TCP), reagent blank control (medium), negative control (HDPE material), and positive control (zinc sulfate). The samples were extracted with Eagle’s minimal essential medium (α-MEM; GIBCO BRL, OK, USA) containing 10% fetal bovine serum (GIBCO BRL, USA) at 37 ± 1 °C for 24 ± 2 h. Cell line (NCTC clone 929; ATCC) was cultured in each of the extraction medium, with 5% CO_2_ at 37 °C for 48 h (*N* = 3). A light microscope was used for qualitative morphological grading of the cytotoxicity test findings.

### 2.3. In Vivo Test (Animal Model)

**Figure 2:**
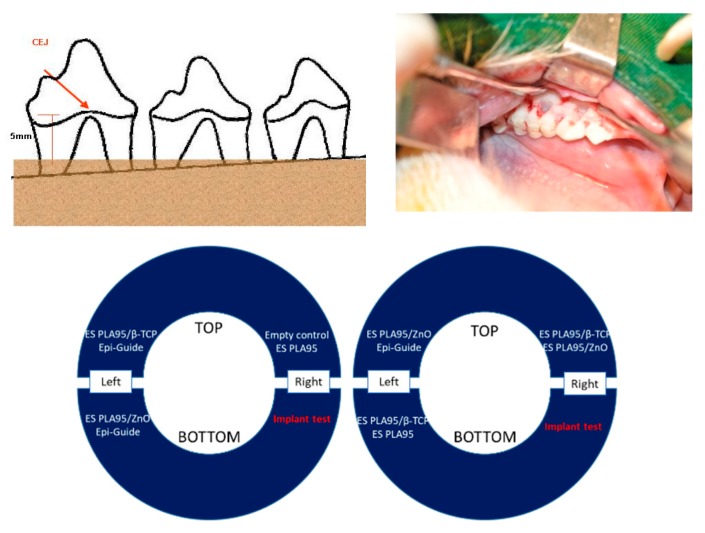
From left to right: Animal model for guided tissue regeneration (GTR) membrane; representative surgery photo and overview of the animal study: the GTR membranes of ES PLA95 and ES PLA95/β-TCP were experimental groups, while the Epi-Guide and empty defect site were used as control groups. Histological and histometric evaluation at week 8 and 16 were performed to determine the healing response of each modality.

Four healthy LanYu pigs (weight: 20–25 kg) were used for animal studies. The protocol was approved by Taipei Medical University (No. LAC-99-0087). Buccal mucoperiosteal flaps were reflected in the bilateral mandibular premolar and molar areas. Buccal alveolar bone was reduced to a level 5-mm apical to the cement–enamel junction (CEJ). The root surface was denuded of the periodontal ligament (PDL) and cementum (CE), and notches were placed at the bone level of each root as in Figure 2. The ES PLA95/β-TCP and control membranes were placed on individual bone defect areas without bone grafting. Flaps were positioned and sutured. All LanYu pigs were sacrificed at the designated times after surgery. Histological and histometric evaluation at 8 and 16 weeks were performed after surgery respectively, to determine the healing response of each treatment modality. Hematoxylin and eosin stain (H&E) staining of the demineralized animal sections were evaluated under a light microscope (40×), and the CE and bone height were measured using the Image J software (NIH, MD, USA).

### 2.4. Clinical Trial

A commercially available PLA dental membrane (PLA; Epi-Guide^®^, Kensey Nash Corp., Exton, PA, USA) was purchased as a control group; while the hydrophilic ES PLA95/β-TCP membranes were used as the experimental group. The protocol was approved by Taipei Medical University Joint Institutional Review Board (TMU-IRB) (No. 201105011)**.** Fifteen periodontal patients with 20 defects were enrolled in the study. The exclusion criteria were patients with unstable vital signs, pregnant women, infection with oral ulcer, participation in other clinical trial, and systemic disease such as leukemia, aplastic anemia, and diabetes. The inclusion criteria were class II or class III furcation defect or intrabony defects with probing depth ≥4 mm in the vertical direction in need of periodontal surgery at the Dental Department of Wan-Fang Hospital. All patients selected for inclusion in the study received a comprehensive periodontal examination and radiographs. The patients were assigned randomly to the control and test groups by the single-blind method. The procedure involved open-flap surgery, scaling and root planning, and additive bone-grafting (particle size 250–500 µm; BonaGraft™, BioTech One Inc., New Taipei City, Taiwan). The GTR membranes were used on the defect sites, the flap was sutured, and patients were instructed on oral health. After 1 week of the surgery, the patients were recalled for adjustment and evaluation and scheduled for follow-up every 4 weeks. Clinical indices such as probing depth (PD), plaque index (PI), gingival index (GI), bleeding on probing (BOP), gingival recession (GR), mobility (MOB), and clinical attachment level (CAL) were assessed 6 months after the surgery.

### 2.5. Statistical Analysis

All data are expressed as mean ± standard deviation (SD). For the in vivo test, the Student’s t-test was used. In the clinical trial, the clinical indices (PD, PI, GI, BOP, GR, MOB, and CAL) were compared by Wilcoxon signed-rank test. Statistical differences between the control and test groups was analyzed by Mann–Whitney U test, and differences were considered statistically significant when the *p*-value was <0.05.

## 3. Results and Discussion

### 3.1. Morphology

**Figure 3:**
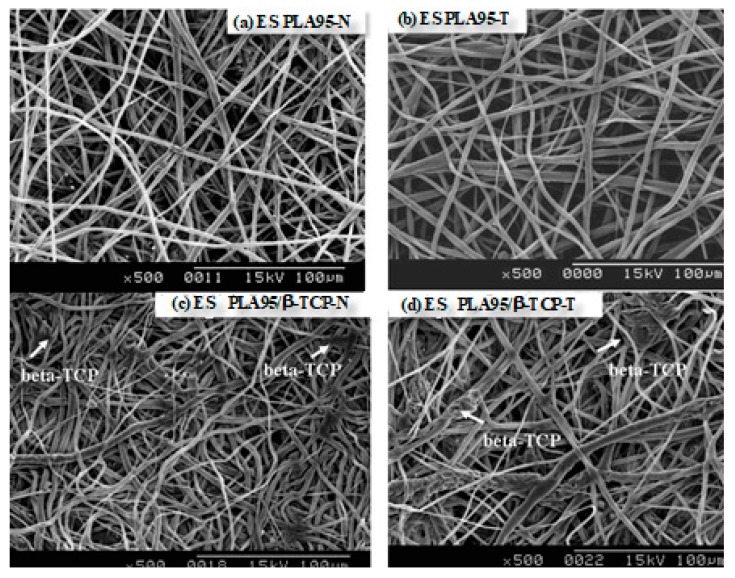
Scanning electron micrographs of the four electro-spun fibrous membranes: (**a**) ES PLA95-N, (**b**) ES PLA95-T, (**c**) ES PLA95/β-TCP-N and (**d**) ES PLA95/β-TCP-T. (N: Without polyethylene oxide (PEO) dip-coating treatment, T: PEO dip-coating treatment). Images courtesy of [22].

The morphology of ES PLA95/β-TCP fibrous membranes are shown in Figure 3. The average fiber diameter for this ES PLA95/β-TCP membrane was 2.37 ± 0.86 µm. The ES PLA95/β-TCP membranes were prepared using dip-coating technique with a dimension of 3.0 × 4.0 cm^2^ (width × length), thickness of 0.3–0.4 mm, suture pull-out force of >200 gf, average porosity of 53.0 ± 4.5%, and average pore size of 25.0 ± 1.0 µm. Unlike the ES PLA95/β-TCP-N with contact angle of 122.6 ± 0.1°, the PEO (polyethylene oxide) dip-coated ES PLA95/β-TCP-T revealed the contact angle of 50.7 ± 0.2° [22]. The hydrophilic surface would help cell adhesion to prevent the membrane expose and avoid infection during healing process.

The typical criteria for ideal GTR membranes are known as cell-occlusive, space making, tissue integrative, clinically manageable and biocompatible [23]. Among the fabrication processes electrospinning, a versatile physical processing technology that does not affect the inherent material properties, has substantially more advantages to manufacture membranes for biomedical and tissue-engineering applications due to their high surface area-to-volume ratio, porosity, and three-dimensional (3-D) structure to mimic an extracellular matrix for enhancing cell-surface interactions [24]. The fiber morphology and diameter of electro-spun poly lactic acid (PLA) fibers were mainly affected by solution properties and process parameters [25,26]. In recent years, growth factors [27,28,29], doxycycline [30,31], and bio-ceramic materials [32,33] have been incorporated into the GTR membranes to improve bioactivity and antibacterial properties. Hydroxyapatite (HAp), β-tricalcium phosphate (β-TCP), and calcium sulfate (CaSO_4_), are osteo-conductive bio-ceramics additives that are widely used in orthopedic and dental applications.

The control group chosen in this study is Epi-Guide^®^, a porous three-layer self-supporting poly-d,l-lactic acid (DL-PLA) barrier for up to 20 weeks with complete bio-resorption between 6–12 months. The Epi-Guide^®^ barrier is claimed to be a hydrophilic membrane that quickly absorbs blood fluid and supports healthy clot formation to maintain gingival flap viability and coverage.

### 3.2. Cytotoxicity Test

As a surgical implant, it is important to verify the biocompatible properties of the ES PLA95/β-TCP membrane. In a previous study, the results indicated that the MC-3T3-E1 cells could adhere and proliferate on the surface of ES PLA95/β-TCP membrane [22]. Therefore, we alternatively evaluated the cytotoxicity of the ES PLA95/β-TCP membrane to ensure the safety of the manufacturing process as well as the PLA95 material. In this study, the MEM elution assay was used to verify the cytotoxicity of the ES PLA95/β-TCP membrane in accordance with the ISO-10993-5 guideline. The results revealed no cell lysis in the membrane extract, reagent blank, and the negative control extracts under light microscope. Thus, it was inferred that the ES PLA95/β-TCP membrane did not exhibit cytotoxic reactivity (Table 1).

### 3.3. In Vivo Test (Animal Model)

**Figure 4:**
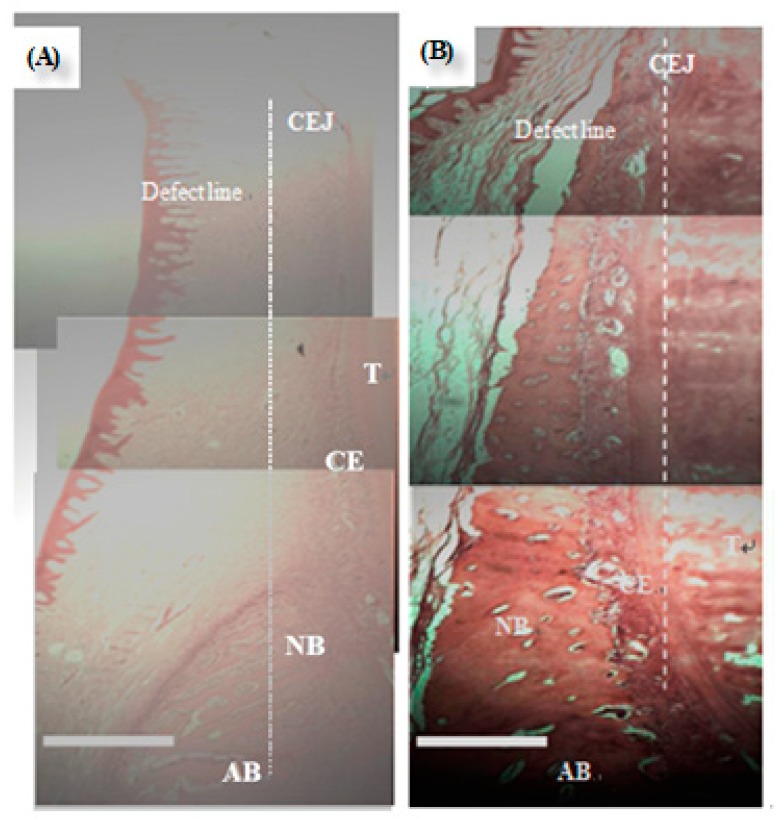
The GTR animal model for cementum and bone height evaluation of (**A**) empty control group and (**B**) representative experimental group. NB: New bone, T: Tooth, AB: Alveolar bone, CE: Cementum, CEJ: Cementoenamel junction. White scale bar noted as 1 mm. Time of implantation: 8 weeks.

There were no severe inflammations and swellings at the flaps in the defect. The periodontal tissues were healthy on the day of sacrifice. The GTR animal model for cementum and bone height were H&E stained and observed under a light microscope (Figure 4).

**Figure 5:**
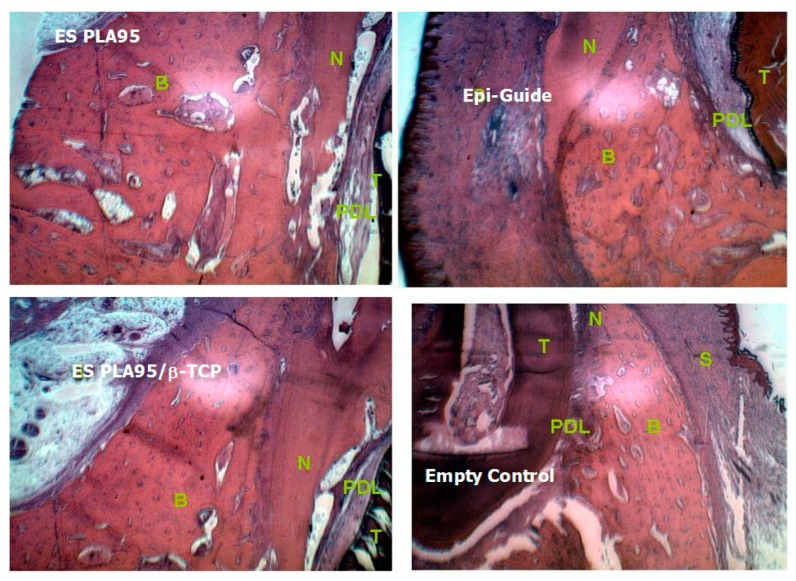
The 8-week H&E stain of various ES PLA95/β-TCP GTR barriers (H&E Stain, 40X) T: Tooth, B: Cementum, N: New bone, PDL: Periodontal ligament.

**Figure 6:**
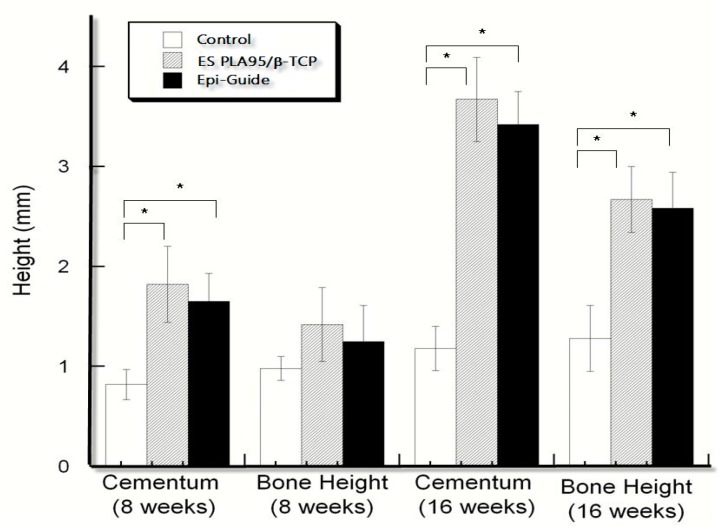
The cementum and bone height of ES PLA95/β-TCP and commercial membranes were measured in LanYu pigs after 8 and 16 weeks. *Differences were considered statistically significant when *p* < 0.05.

The histological results of various 8-week ES PLA95/β−TCP GTR membranes were shown in Figure 5. The histometric evaluation were carried out and listed in Table 2 and Figure 6. The cementum height of the test and control membranes was significantly different between the empty defect after 8 and 16 weeks (*p* < 0.05). The results of bone height showed difference only at 16 weeks (*p* > 0.05) with respective test and control values of 2.67 (±0.33) mm and 2.58 (±0.15) mm; these values were significantly different from the corresponding value of the empty group at 16 weeks (*p* < 0.05). The ES PLA95/β-TCP membrane was effective to block the migration of fast-growing connective tissue into the defect area and in creating some space for the regeneration of new tissues.

In a typical GTR animal study, the usage of bone grafts usually interferes with the efficacy of the GTR membrane [34,35]. Therefore, we intentionally adopted the similar procedure but without bone grafts to confirm the blocking function of the proposed GTR membrane in this study. The results, however, still showed significant differences between the experimental and empty control groups. In addition, few previous studies have shown more bone formation after GTR as compared to that in the empty control without membrane [36,37]. In this study, the cementum height of the experimental group was larger than that of the empty control group without bone grafts at eight weeks. From these results, we conjectured that the addition of bone graft would affect the growth of new bone tissues. The ES PLA95/β-TCP membrane and Epi-Guide^®^ groups were all with similar PLA materials and mechanical properties, with a limited amount of β-TCP content for buffering properties. Therefore, the osteoconductive effect on tissue growth was not significantly different from ES PLA95/β-TCP membrane and Epi-Guide^®^ groups.

### 3.4. Clinical Trial

**Figure 7:**
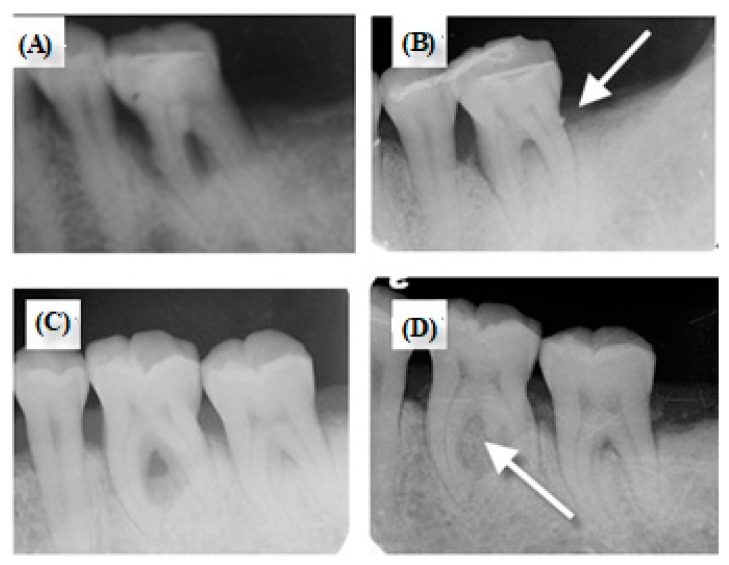
The patient A is with (**A**) pre-surgical radiograph (Tooth No. 36), (**B**) post-surgical radiograph with Epi-Guide^®^ membrane after 6 months. The patent B is with (**C**) pre-surgical radiograph (Tooth No. 37), (**D**) post-surgical radiograph with ES PLA95/β-TCP membrane after 6 months. Arrow (white) indicates the bone regeneration.

All patients selected for inclusion in the study received a comprehensive periodontal examination and radiographs. The study patients were comprised of seven men and eight women with an age range of 35-65 years (mean age: 53 years). Among the seven patients who used Epi-Guide^®^, two patients collected more than one site due to their severity in periodontal disease (7 + 2 = 9 results). Among the eight patients who used ES PLA95/β-TCP GTR membrane, three patients collected more than one site due to their severity in periodontal disease (8 + 3 = 11 results). In patient response, four patients of the control group had a sore tooth at the surgical sites six months after the surgery, while the ES PLA95/β-TCP membrane group did not. In clinical observation, ES PLA95/β-TCP membrane did not show early exposure, implying that the hydrophilic membrane might help gingival tissue adhesion. Six months after the surgery, the clinical indices of each site were measured and re-recorded, such as PI, GI, PI, GI, BOP, PD, GR, MOB, and CAL. Furthermore, pre-surgery and post-surgery radiographies were observed, and reconstruction of bony defect was compared (Figure 7). Several new bone tissues were detected in the defect, indicating that the surgery had good bone-material compatibility outcome.

The clinical indices for the commercial Epi-Guide^®^ group in PD, GI, BOP, and CAL, showed statistically significant differences (*p* < 0.05; Table 3) after treatment, while the experimental group of the ES PLA95/β-TCP membrane showed statistically significant differences for PD, GI, GR, and CAL indices (*p* < 0.05; Table 4) after treatment. The results showed significantly more attachment gain (Epi-Guide^®^, 2 mm; PLA95/β-TCP GTR group, 3 mm; *p* =0.28) and shallower probing depths (Epi-Guide^®^, 3.3 mm; PLA95/β-TCP GTR group, 2.25 mm; *p* =0.85) than the empty control group. The change of clinical indices indicated direct improvement of periodontal inflammation and the efficacy for both the Epi-Guide^®^ and the ES PLA95/β-TCP GTR membrane groups. However, these results did not show statistically significant differences between them (Table 5).

In the clinical study, a few patients of the control group showed soreness at the surgical sites 6 months after the surgery, while the ES PLA95/β-TCP membrane did not. We conjectured that the small amount of β-TCP in this membrane acts as a buffer to reduce the acid releasing during the hydrolysis of ES PLA95 membrane. Therefore, the ES technique is suitable for manufacturing the ES PLA95/β-TCP GTR membrane.

## 4. Conclusions

In this study, ES PLA95/β-TCP membranes were prepared by ES technology. Their effectiveness and safety with regards to cytotoxicity, in vivo animal, and clinical studies were investigated. The ES PLA95/β-TCP membrane did not show cytotoxicity, nor did it result in any inflammation. Significant difference was observed in cementum and bone height before and after surgery using the ES PLA95/β-TCP membrane in animal study. Furthermore, the ES PLA95/β-TCP membrane have a hydrophilic property would prevent early exposure and healing efficacy in this study. In intrabony defects, the use of Epi-Guide^®^ or ES PLA95/β-TCP membranes in GTR procedures yielded comparable clinical results in reducing the probing depth and increasing attachment gain for periodontal patients. The results extended the data bank of resorbable polymer for medical applications where contradictory use of current commercial solution due to clinical condition/preexisting condition of patients

## Figures and Tables

**Table 1:** Cytotoxicity assay of ES PLA95/β-TCP membrane by indirect method.

Extracts of Test Item and Controls	Observation	Grade	Reactivity
Membrane extract, reagent blank, and negative control extract (*N* = 3)	Discrete intracytoplasmic granules; no cell lysis; no reduction of cell growth	0	None
Positive control (*N* = 3)	Complete destruction of the cell layers	4	Severe

Note: (Grade 0: no cell lysis or reduction of cell growth, Grade 1: not more than 20% of the cells are round, Grade 2: not more than 50% of the cells are round and devoid of intra-cytoplasmic granules, Grade 3: not more than 70% of the cell layers contain rounded cells or are lysed, Grade 4: nearly complete or complete destruction of the cell layers).

**Table 2:** The GTR model results of cementum and bone height measurements with empty control and ES PLA95/β-TCP membranes in LanYu pigs after 8 and 16 weeks.

Time	Membranes	Cementum Height (Mean ± SD) mm	Bone Height (Mean ± SD) mm
8 weeks	Empty control	0.82 ± 0.15	0.98 ± 0.12
	ES PLA95/β-TCP	1.82 ±0.38	1.42 ± 0.37
	Epi-Guide^®^ membrane	1.65 ± 0.28	1.25 ± 0.36
16 weeks	Empty control	1.18 ± 0.22 *^,#^	1.28 ± 0.33 *^,#^
	ES PLA95/β-TCP	3.67 ± 0.42 *	2.67 ± 0.33 *
	Epi-Guide^®^ membrane	3.42 ±0.33 ^#^	2.58 ± 0.36 ^#^

*^,^^#^: Statistical significant differences when *p* < 0.05. Values are reported as mean (SD) (*N* = 3).

**Table 3:** Clinical indices before and after treatment for Epi-Guide^®^ group by Wilcoxon signed-rank test.

Clinical Index	N	Before	After	*p*-Value
		Median (Q1–Q3)		
Probing depth (PD) mm	9	6.3 (6–7)	3 (2.7–4.5)	0.0039 *
Plaque index (PI)%	9	16 (10–16)	16 (16–16)	0.62
Gingival index (GI)	9	1 (1–1)	0.5 (0.5–0.5)	0.015 *
Bleeding on probing (BOP)%	9	17 (16–33)	0 (0–16)	0.031 *
Gingival recession (GR) mm	9	0 (0–0)	2 (0–2)	0.25
Mobility (MOB)	9	0 (0–1)	0 (0–1)	1.00
Clinical attachment level (CAL = PD + GR) mm	9	7 (7–8)	5 (4–5)	0.0039 *

*: Differences were considered statistically significant when *p* < 0.05.

**Table 4:** Clinical indices before and after treatment for ES PLA95/β-TCP group by Wilcoxon signed-rank test.

Clinical Index	N	Before	After	*p*-Value
		Median (Q1–Q3)		
Probing depth (PD) mm	11	5.75 (5–8)	3.5 (3–5)	0.001*
Plaque index (PI)%	11	16 (10–16)	10 (0–16)	0.125
Gingival index (GI)	11	1 (1–1)	0.5 (0–0.5)	0.0098*
Bleeding on probing (BOP)%	11	17 (16–50)	16 (0–16)	0.1250
Gingival recession (GR) mm	11	0 (0–2)	2 (1–3)	0.0078*
Mobility (MOB)	11	0 (0–1)	0 (0–1)	0.7500
Clinical attachment level (CAL = PD + GR) mm	11	8 (6–9)	5 (5–8)	0.0020*

*: Differences were considered statistically significant when *p* < 0.05.

**Table 5:** Itemized clinical index differences between the Epi-Guide^®^ and ES PLA95/β-TCP groups after treatment.

Clinical Index	Epi-Guide^®^ Group				ES PLA95/β-TCP Group				*p*-Value
	N	Median	Q1	Q3	N	Median	Q1	Q3	
Probing depth (PD) mm	9	2	3.7	2	11	2.5	3	1.5	0.85
Plaque index (PI)%	9	0	0	0	11	0	16	0	0.33
Gingival index (GI)	9	0.5	0.5	0.5	11	0.5	0.5	0.5	0.24
Bleeding on probing(BOP) %	9	16	17	0	11	0	34	0	0.60
Gingival recession (GR) mm	9	0	0	2	11	1	0	2	0.31
Mobility (MOB)	9	0	0	0	11	0	0	0	1.00
Clinical attachment level (CAL = PD + GR) mm	9	2	4	2	11	2	3	1	0.28

Mann–Whitney *U* test, *: Differences were considered statistically significant when *p* < 0.05.
